# Editorial: Community series in SARS-CoV-2 variants, B lymphocytes, and autoreactivity, volume II

**DOI:** 10.3389/fimmu.2026.1775058

**Published:** 2026-02-06

**Authors:** Marko Z. Radic, Moncef Zouali

**Affiliations:** 1 Department of Microbiology, Immunology and Biochemistry, University of Tennessee Health Science Center, College of Medicine, Memphis, TN, United States; 2 Graduate Institute of Biomedical Sciences, China Medical University, Taichung City, Taiwan

**Keywords:** autoimmunity, B lymphocyte, COVID-19, immune memory, vaccination

## Introduction

There are credible signs that COVID−19 is surging again in certain parts of the world, despite the fact that many countries have reduced surveillance and mandatory reporting, implying that the case numbers are likely under-estimated. In this context, the emergence of SARS-CoV-2 variants shapes the antigenic landscape faced by the human immune system, repeatedly re-selecting suitable responses. Whereas most SARS-CoV-2 viruses trigger protective B-cell responses, a subset of subjects exhibit deregulated B-cell activation, including responses marked by extrafollicular B cell expansion, impaired germinal center formation, and autoreactivity. These observations suggest that a breakdown in immune tolerance, possibly mediated by pro-inflammatory cytokines and TLR stimulation, may activate autoreactive B-cell clones. Given the rapid appearance of SARS-CoV-2 variants with potential immune-evasive mutations, repeated activation of B cells may increase the risk of tolerance disruption.

It is estimated that approximately 10-20% of subjects who have been infected with SARS-CoV-2 will develop long COVID19. However, there is little understanding of what causes this condition. It could be due to persistence of the SARS-CoV-2 virus in the organism or to variants exhibiting an abundance of spike mutations. Additionally, several autoantibodies have been associated with long COVID occurrence, symptoms, and severity (1–4). This Research Topic Community Series aims to provide further insight into how variant-driven antigenic stimulation skews B cell fates toward pathological trajectories, affects the balance between effective immunity and immune-tolerance, and promotes self-reactivity.

## B lymphocytes during SARS-Cov2 infection and re-infection

B cells play several key roles in the immune response to SARS-CoV-2. B cells produce neutralizing antibodies that bind to the virus and block its entry into human cells and reduce viral replication. B cells act as antigen-presenting cells to T helper cells, and this B–T cell collaboration is essential for strong, durable immunity. During infection, B cells undergo affinity maturation in germinal centers, a process that improves antibody affinity and helps maintain durable, potent antibody responses. Following infection or vaccination, B lymphocytes give rise to memory B cells that can quickly produce antibodies during a future exposure to the virus, providing lasting immunity and/or faster recovery. In severe cases, hyperactive or exhausted B cell responses are present, and a subset of B cells, termed, age-associated B cells or extrafollicular B cells, may become overactive and contribute to inflammation. In this Research Topic Community Series, the role of B cells during SARS-CoV-2 reinfection is discussed (Chen et al.).

## Primary immunodeficiencies and their relationship to COVID-19

Studies of inborn errors of immunity (IEI) can lead to refinements of clinical and immunological characteristics of diseases and novel therapeutic approaches. Since 2020, several research groups have identified monogenic defects that predispose individuals—often previously healthy—to life-threatening COVID-19 pneumonia. Genetic defects that increase the risk for severe COVID-19 include the type I Interferon (IFN-I) pathway (TLR3, TLR7, IRF7, IRF9, IFNAR1, IFNAR2, UNC93B1, TICAM1, and TBK1). Mutations or loss-of-function variants in these genes can lead to uncontrolled viral replication and severe disease. Paradoxically, some IEIs, like XLA, result in moderate or mild disease. Identification of highly conserved SARS-CoV-2 targets that are highly immunogenic, but do not elicit auto-reactivity, represents a key step in designing anti-COVID-19 vaccines. In this Research Topic Community Series, scanning the entire spike amino acid sequence for linear epitopes that meet these requirements led to identification of multiple linear epitopes and a highly conserved region that elicits potentially pathogenic autoantibodies (Diaz et al.). Investigation of the humoral response of COVID-19 patients with IEI enabled detection of IgG and IgM antibodies against the SARS-CoV-2 spike protein, and the presence of specific receptor-binding domain-positive (RBD+) IgG+ memory B cells after vaccination against SARS-Cov2 (Molina et al.). These observations could help developing safer pan-coronavirus vaccines.

## SARS-CoV-2 mimicry motifs and auto-reactivity risk

Research has identified several SARS-CoV-2 proteins and epitopes that elicit strong adaptive immune responses, remain highly conserved across variants, and exhibit minimal similarity to human proteins—reducing the likelihood of molecular mimicry and autoimmune cross-reactivity. Regions reported to be highly conserved across variants, immunogenic, and not associated with significant auto-reactivity have been described in the nucleocapsid (N) Protein, the S2 Subunit of the Spike (S) protein, membrane (M) protein, non-Structural Proteins (NSPs) involved in replication, and ORF3a. Epitopes most associated with concern are located primarily in certain regions of Spike S1, and parts of N. By contrast, S2, M, and essential NSPs show minimal homology to self peptides, no strong associations with post-COVID-19 autoimmune sequelae, and their structures are stable across variants, probably because of reduced pressure for immune escape. In this Research Topic Community Series, investigation of SARS-CoV-2 vaccine-induced immunity indicates a wide heterogeneity in the magnitude of the receptor-binding domain (RBD)-specific neutralizing responses, and illustrate that antibody levels largely depend on prior exposure to SARS-CoV-2, the number of vaccine doses, and the type of vaccine received (Hernández-Luis et al.).

## Rheumatoid factors as bridge between COVID-19 and rheumatoid arthritis

Several published reports have identified autoantibodies whose appearance in COVID-19 patients coincided with the infection. Conversely, it has long been suspected that infections may provide a trigger for autoimmune disease. Researchers have used antigen arrays to screen for many antibody specificities to draw parallels with known targets associated with specific autoimmune diseases. Titi et al. provide the next level of insight in this Research Topic Community Series. They break down the serum reactivity in patients with rheumatoid arthritis (RA) patients during COVID-19 convalescence but without clinical RA, and normal controls. In an important refinement over previous studies, Titi et al. screen for epitopes of immunoglobulin G (IgG) in their native state and containing citrulline or homocitrulline modifications, such as are known targets in RA. As previously shown, most of the epitopes on IgG peptides react with RA sera (5), yet certain peptides, even in their specifically modified form, recognize post-COVID-19 convalescent sera. This observation raises the question whether such reactivities arise independently and are coincidental, or whether they represent an early point at which immune reactivity to self may arise, only to depend on subsequent events to further drive development of autoimmunity. The results could identify the initial divergence from tolerance that may evolve over time into overt autoimmunity. If so, these epitopes may represent a valuable biomarker for a very early transition point toward RA. Further studies will shed light on this possibility.

## Conclusions

The papers in this Research Topic Community Series are excellent examples of research that is made possible by the availability of highly sophisticated and precise experimental tools and the world-wide attention focused on a highly infectious and deadly disease. Because early observations during the pandemic raised the specter of an overlap between chronic autoimmune diseases, susceptibility to severe systemic infections, and suggested a possible trigger in the environment for autoimmunity, important questions were raised that have been debated since the beginning of immunology research. Thus, it is important to highlight the papers in this Research Topic, which shine a critical light on improved virus-specific vaccines, survey the immune landscape of effective responses to infection, and search for risks leading to autoimmunity and immunodeficiency that are laid bare by this pandemic pathogen.

## References

[1] Knight JS Caricchio R Casanova JL Combes AJ Diamond B Fox SE . The intersection of COVID-19 and autoimmunity. J Clin Invest. (2021) 131:1–9. doi: 10.1172/JCI154886, PMID: 34710063 PMC8670833

[2] Woodruff MC Ramonell RP Haddad NS Anam FA Rudolph ME Walker TA . Dysregulated naive B cells and *de novo* autoreactivity in severe COVID-19. Nature. (2022) 611:139–47. doi: 10.1038/s41586-022-05273-0, PMID: 36044993 PMC9630115

[3] Tsay GJ Zouali M . Cellular pathways and molecular events that shape autoantibody production in COVID-19. J Autoimmun. (2024) 147:103276. doi: 10.1016/j.jaut.2024.103276, PMID: 38936147

[4] Wilhelm F Cadamuro J Mink S . Autoantibodies in long COVID: a systematic review. Lancet Infect Dis. (2025) 8:S1473-3099(25)00411-6. doi: 10.1016/S1473-3099(25)00411-6, PMID: 40934937

[5] Mergaert AM Zheng Z Denny MF Amjadi MF Bashar SJ Newton MA . Rheumatoid factor and anti-modified protein antibody reactivities converge on igG epitopes. Arthritis Rheumatol. (2022) 74:984–91. doi: 10.1002/art.42064, PMID: 35001558 PMC9156533

